# Tunable Adhesive Self-Cleaning Coating with Superhydrophobicity and Photocatalytic Activity

**DOI:** 10.3390/nano11061486

**Published:** 2021-06-03

**Authors:** Xuan Wang, Weihua Ao, Sijia Sun, Han Zhang, Run Zhou, Yangzi Li, Jie Wang, Hao Ding

**Affiliations:** Beijing Key Laboratory of Materials Utilization of Nonmetallic Minerals and Solid Wastes, National Laboratory of Mineral Materials, School of Materials Science and Technology, China University of Geosciences, Xueyuan Road, Haidian District, Beijing 100083, China; wangxuan0505@163.com (X.W.); sunsijia@cugb.edu.cn (S.S.); zhanghan0050@163.com (H.Z.); 3003180004@cugb.edu.cn (R.Z.); liyangzi@cugb.edu.cn (Y.L.); 3003170004@cugb.edu.cn (J.W.)

**Keywords:** superhydrophobicity, photocatalytic activity, tunable adhesion, TiO_2_, PDMS

## Abstract

Superhydrophobic coatings with intelligent properties have attracted much attention because of their wide application in many fields. However, there is a limited amount of literature on superhydrophobic coatings whose wettability and adhesion can be adjusted by UV irradiation and calcination at the same time. In this study, amorphous SiO_2_ microspheres (A-SiO_2_) and nano-TiO_2_ particles (N-TiO_2_) were used to fabricate A-SiO_2_/N-TiO_2_ composites by wet grinding, and then, they were modified with polydimethylsiloxane (PDMS) and sprayed onto substrate surfaces to obtain a tunable adhesive superhydrophobic A-SiO_2_/N-TiO_2_@PDMS coating. It is worth noting that the wettability and adhesion of the coating to water droplets could be adjusted by UV irradiation and calcination. The mechanisms of the aforementioned phenomena were studied. Moreover, methyl orange solution could be degraded by the coating due to its photocatalysis. The as-prepared coating had good adaptation to different substrates and outdoor environments. Moreover, the surfaces of these coatings exhibited the same liquid repellency towards different droplets. This research provides an environmental strategy to prepare advanced self-cleaning coatings.

## 1. Introduction

The “lotus effect” [1] and the “rose effect” [2] are the two most representative superhydrophobic biological phenomena in nature. Both lotus leaf and rose petal surfaces are superhydrophobic. However, lotus leaf surfaces have low adhesive forces to water droplets, which means water can roll off them easily and remove the dust attached to their surfaces. This is defined as the “lotus effect”. Unlike lotus leaf surfaces, the rose petal surfaces have high adhesive forces to water droplets, and they cannot roll off their surfaces easily. The study of these phenomena can not only reveal the scientific principles but also solve some engineering problems through practical application. Superhydrophobic surfaces such as lotus leaf surfaces can be used for self-cleaning coatings [3,4], oil–water separation [5,6], anti-corrosion [7], and anti-icing [8]. Meanwhile, superhydrophobic surfaces such as rose petal surfaces can be used for the no-loss transport of microdroplets [9,10] in biology or medicine.

Apart from dust, organic pollutants often attach to the surfaces of building exterior walls. It is difficult to remove the organic pollutants only by the rolling of water droplets from the coating surfaces, even if they are lotus-like self-cleaning coatings. Moreover, the accumulation of these organic pollutants results in the loss of superhydrophobicity [11]. Fortunately, the organic pollutants can be degraded effectively by the photocatalytic activity of some semiconductors. Therefore, the advanced superhydrophobic self-cleaning coating should also have photocatalytic activity. Among these semiconductors, TiO_2_ has the advantages of excellent photocatalytic activity, chemical inertness, and non-toxicity [12,13,14,15]. In recent years, it has attracted great interest in the preparation of superhydrophobic materials with photocatalytic activity using TiO_2_. Parkin [16] et al. prepared a superhydrophobic film with photocatalytic activity through direct incorporation of TiO_2_ into a PDMS by aerosol-assisted chemical vapor deposition. However, the superhydrophobic film must be based on PDMS, which does not have good adaptability to other substrates. Seeger [17] et al. prepared a superhydrophobic film with TiO_2_ nanoparticles, silicone nanowires, and polyethylene. The static contact angle of its surfaces reached 168°, and the organic pollutants could be degraded under UV irradiation. Nevertheless, the preparation process is complicated, harsh, time consuming, and not suitable for large-scale applications. Lyons [18] et al. reported a multifunctional TiO_2_/high-density polyethylene nanocomposite surface. The static contact angle of the surface reached 158°, but it decreased to 120° after UV irradiation. This showed that the coating cannot maintain superhydrophobicity under UV irradiation. Therefore, it could not be used outdoors. Wu [19] et al. fabricated a self-healing superhydrophobic coating with polystyrene, fluorine compounds, and fluorine compound-modified SiO_2_ and TiO_2_. However, not only are fluorides expensive, but they are also harmful to the environment, which limits their large-scale application [20,21].

Herein, N-TiO_2_ was loaded onto A-SiO_2_ surfaces by the hydrophobic agglomeration method. Then, A-SiO_2_/N-TiO_2_ composite particles were modified with PDMS. Finally, the modified A-SiO_2_/N-TiO_2_ composite particles were sprayed onto substrate surfaces, and the A-SiO_2_/N-TiO_2_@PDMS coatings with superhydrophobicity and photocatalytic activity were formed after drying. The wettability of the coating could be adjusted by UV irradiation and calcination. Furthermore, the as-prepared coating had good adaptability to different types of substrates, and methyl orange could be degraded by A-SiO_2_/N-TiO_2_@PDMS under UV irradiation. Moreover, the coating had the advantages of environmental protection (no organic solvent, such as hexane or toluene), low cost, and powerful operability. Therefore, the coating has a wide range of applications.

## 2. Materials and Methods

### 2.1. Materials

A-SiO_2_ was supplied by Jiaozuo Fluoride New Energy Technology Co., Ltd. (Jiaozuo, Henan, China). The N-TiO_2_ used in this study was commercially available. Polydimethylsiloxane was provided by Aladdin Reagents Co., Ltd. (Shanghai, China). Sodium oleate and grade sodium stearate were purchased from Beijing Chemical Works (Beijing, China). Sulfuric acid (H_2_SO_4_) and sodium hydroxide (NaOH) used in the experiments were supplied by Beijing Chemical Industry Group Co., Ltd. (Beijing, China). They were all analytical agents. Deionized water and ethanol were also used.

### 2.2. Preparation Methods

**Figure 1:**
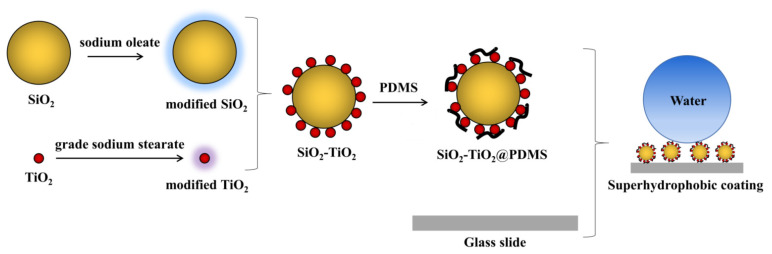
Preparation flowchart of superhydrophobic A-SiO_2_/N-TiO_2_@PDMS coating.

The preparation process of the A-SiO_2_/N-TiO_2_@PDMS coating is shown in Figure 1. The first step was the preparation of the A-SiO_2_/N-TiO_2_ composites with the hydrophobic aggregation method [22]. In brief, 5 g of A-SiO_2_ and 20 g of N-TiO_2_ were added into two beakers, which were dispersed by adding 10 times the mass of distilled water under continuous agitation at 50 °C. Then, 0.05 g of sodium oleate and 0.2 g of sodium stearate were dissolved in a small amount of water at 50 °C. The sodium oleate and sodium stearate solutions were added to A-SiO_2_ and N-TiO_2_ suspensions, respectively, and they were stirred for 1 h. Finally, the modified N-TiO_2_ suspension was added dropwise to the modified A-SiO_2_ suspension. After stirring for 90 min at 50 °C under pH = 9, the A-SiO_2_/N-TiO_2_ suspension was obtained, which was dried at 105 °C, and then the A-SiO_2_/N-TiO_2_ composites were obtained.

In the second step, the superhydrophobic A-SiO_2_/N-TiO_2_@PDMS coating was fabricated by the spraying method, which includes the following steps: 10.0 g ethanol, 2.0 g A-SiO_2_/N-TiO_2_ composites, and 0.5 g PDMS are added to a beaker; subsequently, they are stirred for 30 min and sprayed onto the substrate surfaces; finally, the superhydrophobic coating is obtained after drying at 60 °C.

### 2.3. Characterizations

Water contact angles (WCAs) were used to evaluate the wettability of the surfaces. Contact angle hystereses (CAHs) and the surface adhesive force to water droplets were used to evaluate the adhesion of the surfaces to water droplets. The experiments of photocatalytic degradation were performed, and methyl orange solution was used as the degradation object. The detailed description of the aforementioned measurements and characterizations can be seen in the Supporting Information.

## 3. Results and Discussion

### 3.1. Wettability and Adhesion

**Figure 2:**
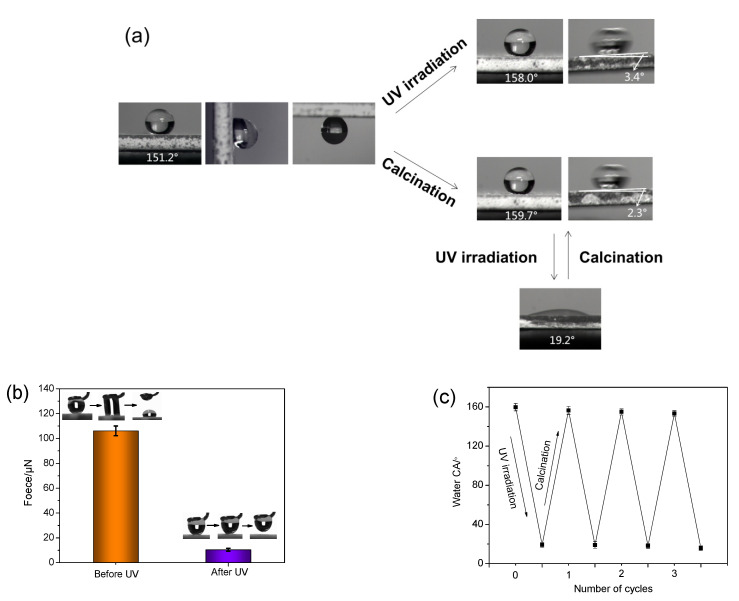
(**a**) The changes in the coating’s wettability and adhesion to water droplets before and after UV irradiation and calcination. (**b**) The adhesion force of the coating to a water droplet before and after UV irradiation. (**c**) The changes in WCAs of the coating after alternate UV irradiation and calcination.

The WCAs, CAHs, adhesion forces to water droplets, and their changes after UV irradiation and calcination were measured. The results are displayed in Table S1 and Figure 2. It was observed that the WCA of the coating was 151.2°. After UV irradiation for 1 min and 8 h, the WCAs of the coatings increased to 158.0° and 158.1°, respectively. After calcination at 400 °C for 30 min, the WCA of the coating increased to 159.7°. The results illustrate that the coating had excellent superhydrophobicity, photostability, and thermal stability. Moreover, the thermal stability of the as-prepared coating was further analyzed according to the TG results of A-SiO_2_/N-TiO_2_ and A-SiO_2_/N-TiO_2_@PDMS, which can be seen in the Supporting Information (Figure S1). The adhesion forces of the coating to water droplets are shown in Table S1 and Figure 2b. The adhesion force of the coating to a water droplet was as high as 106.1 μN before UV irradiation, and the water droplet could not roll off its surface. After UV irradiation for 1 min, the adhesion force of the surface to water droplets decreased sharply to 10.4 μN, and the CAH was 3.4°. After calcination at 400 °C for 30 min, the CAH of the coating was 2.3°. It was observed that the coating surface had transformed into a typical lotus-like surface from a rose petal-like surface after UV irradiation and calcination. Due to UV and sunlight irradiation, the environmental conditions that the self-cleaning coatings must face are inevitable, and it is important that the coating maintains the superhydrophobicity and that it adopts low adhesion to water droplets under UV irradiation. This would be beneficial for the as-prepared coating used as an outdoor self-cleaning coating.

The experimental results also exhibited that the calcined coating surface changed from the superhydrophobic state (CA = 159.7°) to the hydrophilic state (CA = 19.2°) after UV irradiation for 2 h. Interestingly, the hydrophilic surface could return to the original superhydrophobic state after calcination at 400 °C for 2 h. Eventually, the coating surface became hydrophilic after three cycles of UV irradiation and calcination (Figure 2c). It was observed that the wettability of the coating could be adjusted by alternating UV irradiation and calcination for a certain number of times.

### 3.2. Self-Cleaning Properties

#### 3.2.1. Self-Cleaning Process and Principle

**Figure 3:**
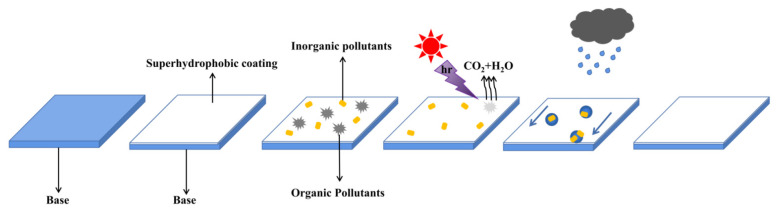
Schematic diagram of the self-cleaning process.

It is suggested that the self-cleaning properties of the coating include the “lotus effect” and photocatalytic activity. The process and principle of the self-cleaning effect are shown in Figure 3, which mainly include three parts: (1) The organic pollutants on the coating surface are degraded by photocatalysis under UV or sunlight. (2) The superhydrophobicity of the coating is further enhanced under UV or sunlight, and its adhesion to water droplets decrease sharply. The surface of the coating becomes a self-cleaning surface like the lotus leaf. (3) When it rains, the raindrops roll off the coating surface and remove the dust attached to the coating surface.

#### 3.2.2. Photocatalytic Degradation and the “Lotus Effect”

**Figure 4:**
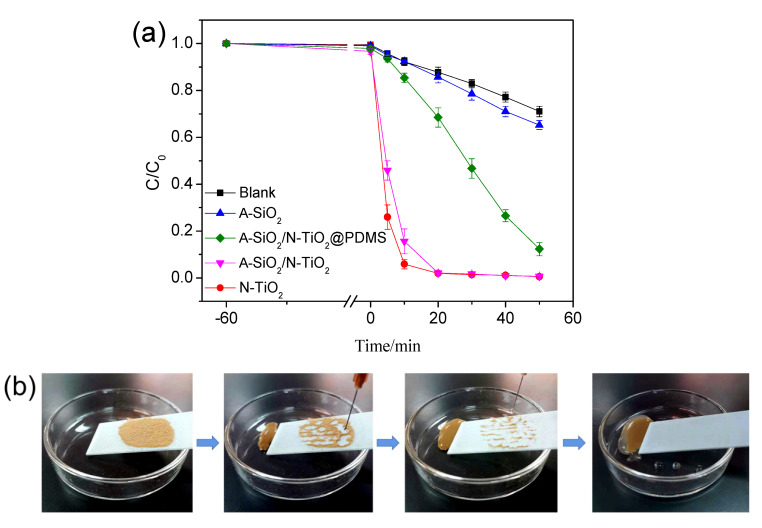
(**a**) Photodegradation of methyl orange by A-SiO_2_/N-TiO_2_@PDMS and other samples; (**b**) simulation experiment of the “lotus effect”.

Figure 4a displays the results of the photodegradation by A-SiO_2_, N-TiO_2_, A-SiO_2_/N-TiO_2_, and A-SiO_2_/N-TiO_2_@PDMS under UV irradiation. The C/C_0_ values of the samples decreased slightly in the stage of dark reaction, indicating that these samples have no adsorption on methyl orange. In the stage of photoreaction, the C/C_0_ value of the dilution added with A-SiO_2_ decreased slightly, demonstrating that A-SiO_2_ had no photocatalytic property. However, the suspensions were decolorized by N-TiO_2_ and A-SiO_2_/N-TiO_2_ in 20 min. Moreover, the C/C_0_ of the suspensions decreased to 0.02, indicating the degradation rate was 98%. This illustrates that N-TiO_2_ and A-SiO_2_/N-TiO_2_ had good photocatalytic degradation activities. However, it was found that A-SiO_2_/N-TiO_2_@PDMS floated on the reaction solution surface and did not fully mix with it because of the superhydrophobicity. Even so, the C/C_0_ of the dilution reached 0.12, illustrating that the degradation rate was 87.7%. Therefore, it is inferred that the as-prepared coating had a photocatalytic effect on organic compounds.

The “lotus effect” of the coating was verified by a simple simulation experiment. Figure 4b presents the experimental process and result. Some dust was spread on its surface, which was irradiated by UV light for 1 min. Then, the coating was placed in a glass dish at a certain angle, allowing for the soil on its surface to be cleaned by the falling water, which is similar to the “lotus effect”.

### 3.3. Liquid Repellency and Adaptability

**Figure 5:**
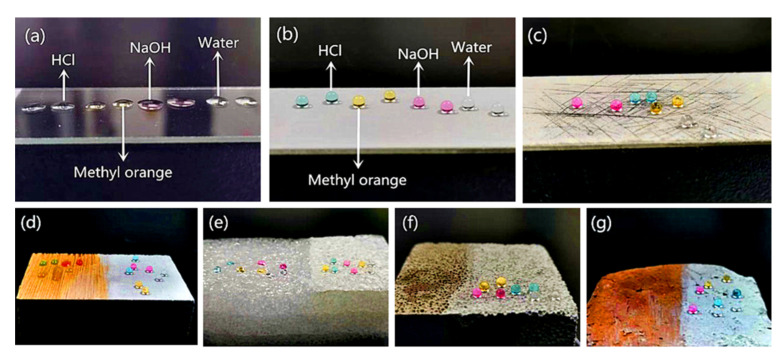
Wettability of the surfaces of (**a**) a glass slide, (**b**) the A-SiO_2_/N-TiO_2_@PDMS coating after UV irradiation, and (**c**) the mechanically damaged A-SiO_2_/N-TiO_2_@PDMS coating after UV irradiation to water, HCl, NaOH, and methyl orange solution droplets, respectively; wettability of the surfaces of (**d**) wood, (**e**) foam, (**f**) concrete, and (**g**) brick before (left) and after (right) being sprayed with A-SiO_2_/N-TiO_2_@PDMS and UV irradiation to water, HCl, NaOH, and methyl orange solution, respectively.

As is well known, there are many types of outdoor substrates, and their surfaces face harsh environments, including contact with different liquids. Therefore, superhydrophobic coatings can be widely used only if they have outstanding adaptability to various substrates while exhibiting liquid repellency and low adhesion to different liquids. To investigate the adaptability of the coating to different substrates, A-SiO_2_/N-TiO_2_@PDMS was sprayed onto the surfaces of wood, foam, concrete block, and brick, and then they were exposed to UV light for 1 min. Furthermore, the wettability of these surfaces to water, NaOH solution, HCl solution, and methyl orange solution was investigated. Figure 5a,b show that the above-mentioned droplets spread almost completely on the glass slide surface, illustrating that the surface of the glass slide was hydrophilic. However, the surface sprayed with A-SiO_2_/N-TiO_2_@PDMS and irradiated by UV light exhibited excellent liquid repellency towards the above-mentioned droplets, and they were spherical in shape on the above-mentioned surfaces. Table S2 shows the WCAs of the surfaces in Figure 5a,b to different droplets. The CAs of a glass slide to water, HCl solution, NaOH solution, and methyl orange solution were 45.6°, 41.2°, 23.5°, and 43.1°, respectively, while the CAs of the surfaces sprayed with A-SiO_2_/N-TiO_2_@PDMS and irradiated by UV light for 1 min were 158.0°, 156.4°, 153.8°, and 155.8°, respectively. Furthermore, the above-mentioned droplets remained sphere on the coating surface when it was mechanically damaged by a knife (Figure 5c). This indicates that the coating had a good mechanical stability. As can be seen from Figure 5d–g, the above-mentioned droplets spread quickly when they were dripping on the surfaces of wood, foam, concrete block, and brick, indicating that the surfaces of these substrates were hydrophilic. However, after being sprayed with A-SiO_2_/N-TiO_2_@PDMS and illuminated by UV light, these surfaces showed superhydrophobic, and the droplets were sphere in shape on these surfaces. The results suggest that the as-prepared coating had good adaptability to different substrates and showed the same liquid repellency towards different liquids. Therefore, the as-prepared A-SiO_2_/N-TiO_2_@PDMS can be used to construct self-cleaning coatings on the surfaces of various substrates.

### 3.4. Durability

**Figure 6:**
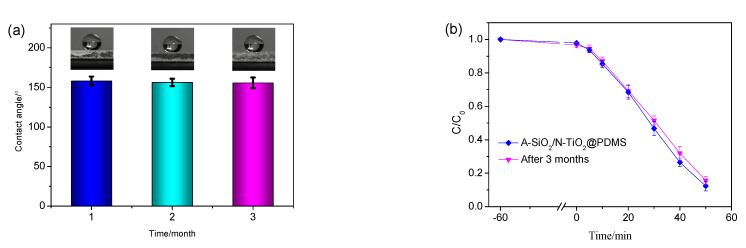
The changes in (**a**) WCAs and (**b**) photocatalytic degradation effects of the A-SiO_2_/N-TiO_2_@PDMS coating after being placed outdoors for 3 months.

To investigate the adaptability and durability of the A-SiO_2_/N-TiO_2_@PDMS coating to the outdoor environment, it was placed outdoor for 3 months. Then, the changes in the WCAs and photocatalytic degradation effect were measured. The results are displayed in Figure 6. The WCA of the as-prepared coating was 155.7° after being placed outdoors for 3 months, which gave a barely measurable reduction. The C/C_0_ value of the methyl orange dilution was 0.16, indicating the degradation rate was 84.4%. An obvious decrease was not observed when compared with the original degradation rate of 87.7%. Therefore, the as-prepared coating has good adaptability to the outdoor environment, and it has the potential to be used outdoors for a long time.

### 3.5. Morphology and Structure

#### 3.5.1. Morphology

**Figure 7:**
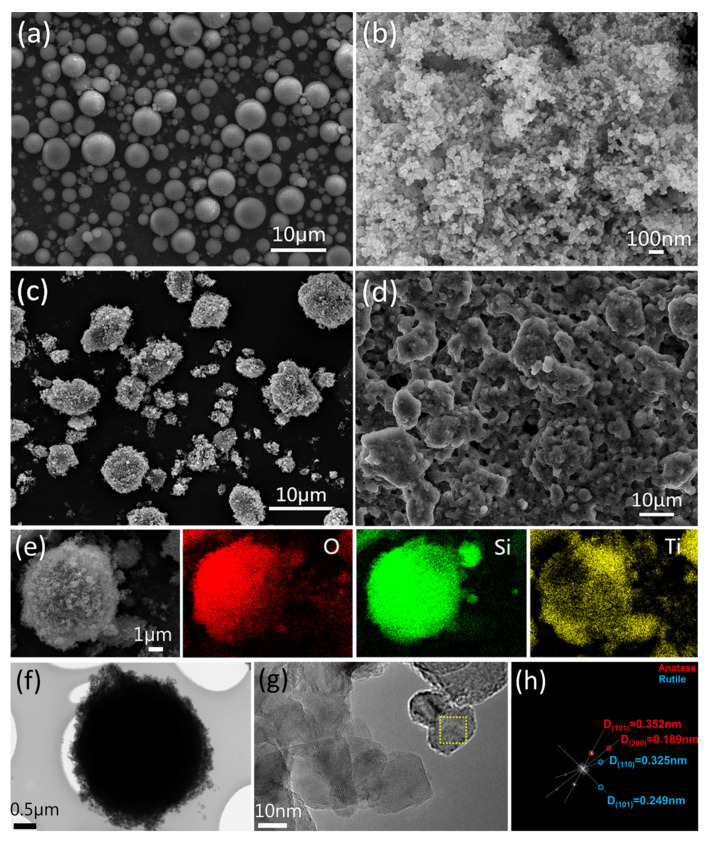
SEM images and EDS element mapping data of A-SiO_2_, N-TiO_2_, and A-SiO_2_/N-TiO_2_. (**a**) SEM image of A-SiO_2_; (**b**) SEM image of N-TiO_2_; (**c**) SEM image of A-SiO_2_/N-TiO_2_; (**d**) SEM image of the A-SiO_2_/N-TiO_2_@PDMS coating surface; (**e**) EDS element mapping images of O, Si, and Ti of A-SiO_2_/N-TiO_2_; (**f**) TEM of A-SiO_2_/N-TiO_2_; (**g**) HRTEM image of A-SiO_2_/N-TiO_2_; (**h**) FFT analysis of a selected area of A-SiO_2_/N-TiO_2_.

To investigate the structures of the samples, SEM was carried out. Figure 7a,b are SEM images of A-SiO_2_ and N-TiO_2_, respectively. Figure 7a shows that A-SiO_2_ was composed of spherical particles dispersed from each other with smooth surfaces, and the particle size was between 0.5 μm and 5 μm. As is shown in Figure 7b, the granular N-TiO_2_ with a particle size of 0.2–0.3 μm was a regular aggregate. Figure 7c,e are SEM images of A-SiO_2_/N-TiO_2_. It can be seen that N-TiO_2_ was uniformly loaded onto the A-SiO_2_ surfaces, and the A-SiO_2_/N-TiO_2_ particles showed good dispersity. The structure of A-SiO_2_/N-TiO_2_ is similar to that of the lotus leaf, which has micro-scale mastoids and nano-scale rods on its surface. 

Figure 7d is an SEM image of the as-prepared coating surface. The A-SiO_2_/N-TiO_2_@PDMS suspension was sprayed on the surface of the substrate to form a honeycomb-like structure. Figure 7e shows the element distribution on the A-SiO_2_/N-TiO_2_ surfaces. It can be seen that O and Si were distributed in the contour range of spherical particles in the scanning area, reflecting the property of A-SiO_2_. Moreover, Ti was evenly distributed in the scanning area and corresponded to the position of A-SiO_2_. It was proved that N-TiO_2_ had been loaded onto the A-SiO_2_ surfaces successfully.

Figure 7f–h are TEM images of A-SiO_2_/N-TiO_2_. The morphology of A-SiO_2_/N-TiO_2_ can be seen in Figure 7f, which shows that the A-SiO_2_ was approximately 3 μm in diameter and was covered by N-TiO_2_. Figure 7g is an enlarged image of Figure 7f. The lattice fringe of N-TiO_2_ can be seen, and the average size of N-TiO_2_ was 20 nm–30 nm. The lattice fringes in the selected area can be transformed into diffraction spots by Fourier transform, and the results are displayed in Figure 7h. The crystal plane spacing of N-TiO_2_ can be divided into two types. The crystal plane spacings of 0.352 nm and 0.189 nm correspond to the (101) and (200) crystal plane of anatase, respectively. The crystal plane spacings of 0.325 nm and 0.249 nm correspond to the (110) and (101) crystal plane of rutile, respectively. The results show that N-TiO_2_ included anatase and rutile, which were consistent with the results of XRD.

#### 3.5.2. Crystalline Phase

**Figure 8:**
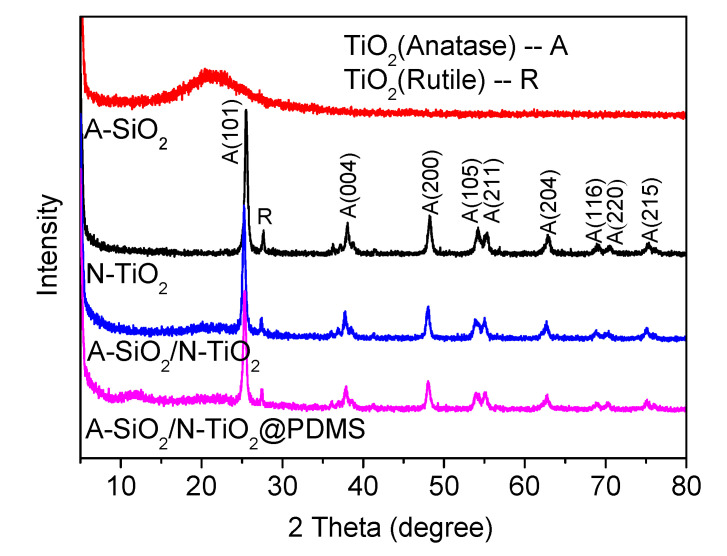
XRD patterns of A-SiO_2_, N-TiO_2_, A-SiO_2_/N-TiO_2_, and A-SiO_2_/N-TiO_2_@PDMS.

The crystalline phases of samples can be characterized by X-ray diffractograms. Figure 8 displays the XRD patterns of samples. It was observed that A-SiO_2_ had a peak of amorphous material around 2θ = 21°, which indicated that the phase of A-SiO_2_ was amorphous. There were several peaks in the diffractogram of N-TiO_2_ corresponding to the crystal planes of anatase and rutile. The results manifested that TiO_2_ was composed of anatase and rutile. In the pattern of A-SiO_2_/N-TiO_2_, the peaks of anatase and rutile were clear, while the peak of amorphous material disappeared. This is because A-SiO_2_ was covered by N-TiO_2_. It was also demonstrated that N-TiO_2_ was loaded on the A-SiO_2_ surfaces successfully. The peak shape of the diffractogram of A-SiO_2_/N-TiO_2_@PDMS was almost the same as that of A-SiO_2_/N-TiO_2_, and no new peak appeared. This is due to the fact that PDMS is an organic polymer, and it has no crystalline phase.

#### 3.5.3. FT-IR Spectrum

**Figure 9:**
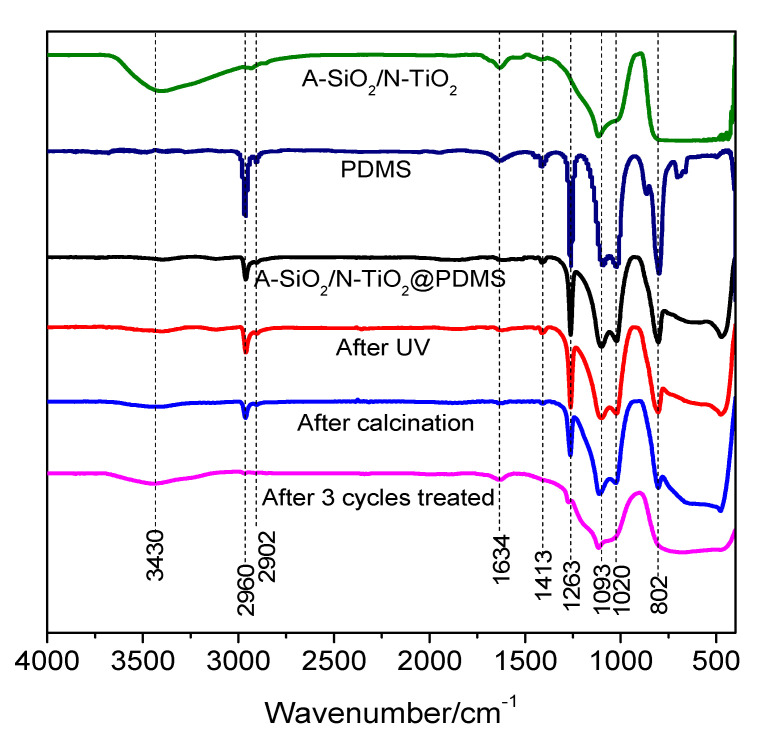
FT-IR spectra of A-SiO_2_/N-TiO_2_, PDMS, A-SiO_2_/N-TiO_2_@PDMS, and A-SiO_2_/N-TiO_2_@PDMS after UV irradiation; A-SiO_2_/N-TiO_2_@PDMS after calcination at 400 °C; and A-SiO_2_/N-TiO_2_@PDMS after 3 cycles of calcination and UV irradiation.

According to the experimental phenomena discussed above, UV irradiation and calcination affected the surface properties of the superhydrophobic coating. Therefore, infrared spectra were used to test the changes in the surface functional groups of the samples. The infrared spectra of samples are presented in Figure 9. The peaks at 2960 cm^−1^, 2902 cm^−1^, 1413 cm^−1^, and 1263 cm^−1^ were caused by the -CH_3_ of PDMS. Moreover, the peak at 802 cm^−1^ can be ascribed to the stretching vibration of Si-O-Si. The above peaks were caused by PDMS. The peaks at 1093 cm^−1^ and 1020 cm^−1^ were caused by the stretching vibration of Si-O. The broad peak at 3430 cm^−1^ can be ascribed to the -OH anti-symmetric stretching vibration peak of structured water, and the peak near 1634 cm^−1^ can be ascribed to the H-O-H bending vibration peak of water.

It can be seen that the bending vibration peaks of -OH at 3430 cm^−1^ and H-O-H at 1634 cm^−1^ were clear in the spectrum of A-SiO_2_/N-TiO_2_. However, the above-mentioned peaks almost disappeared, and the peaks of PDMS could be seen in the spectrum of A-SiO_2_/N-TiO_2_@PDMS. This is because the A-SiO_2_/N-TiO_2_ was covered by PDMS. After UV irradiation, the peak shape did not significantly change, but the -CH_3_ peaks at 2960 cm^−1^ and 1263 cm^−1^ increased slightly, which indicates that the photocatalytic activity of N-TiO_2_ could not decompose PDMS but might result in the rearrangement of hydrophobic groups, and more -CH_3_ appeared on the surface of the coating [23]. After calcination at 400 °C, the characteristic peak of PDMS was weakened, indicating that part of PDMS might be decomposed during the process of calcination at 400 °C. Moreover, the -OH peak at 3430 cm^−1^ was slightly enhanced, indicating that some -OH from A-SiO_2_/N-TiO_2_ was exposed on the surfaces. After three cycles of UV irradiation and calcination, the peaks of PDMS almost disappeared, and the shape of the spectrum was similar to that of A-SiO_2_/N-TiO_2_. This indicates that PDMS was completely decomposed during the cycles of UV irradiation and calcination. Moreover, the bending vibration peaks of -OH at 3430 cm^−1^ and H-O-H at 1634 cm^−1^ increased, indicating that more -OH was exposed on the surface.

#### 3.5.4. XPS Spectra

**Figure 10:**
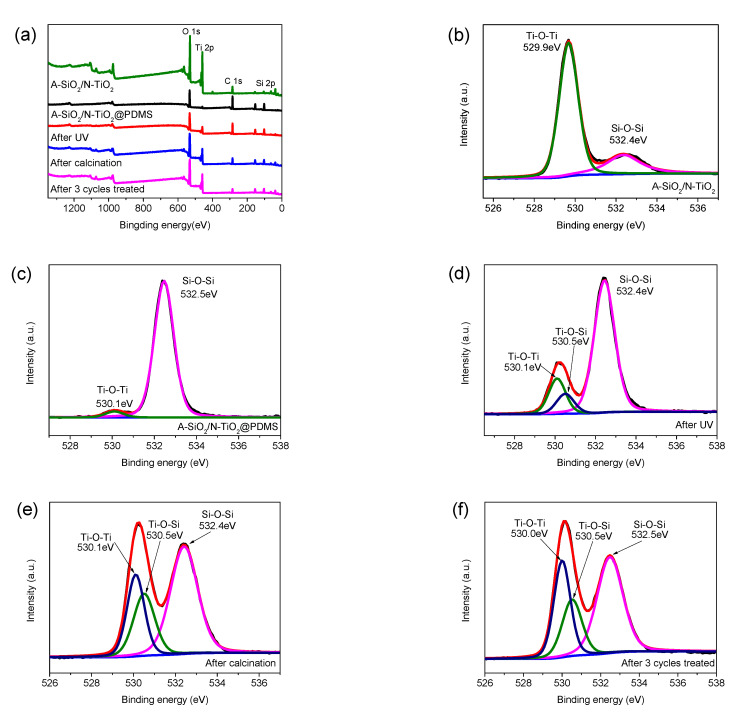
(**a**) Full-scan XPS spectra of A-SiO_2_/N-TiO_2_, A-SiO_2_/N-TiO_2_@PDMS, A-SiO_2_/N-TiO_2_@PDMS after UV irradiation; A-SiO_2_/N-TiO_2_@PDMS after calcination at 400 °C; and A-SiO_2_/N-TiO_2_@PDMS after three cycles of UV irradiation and calcination. (**b**–**f**) Narrow-scan spectra of O 1s in the above spectra.

To further investigate the reactions between PDMS and composite particles, XPS was carried out. Figure 10a shows the full-spectrum XPS scan of A-SiO_2_/N-TiO_2_, A-SiO_2_/N-TiO_2_@PDMS, A-SiO_2_/N-TiO_2_@PDMS after UV irradiation, A-SiO_2_/N-TiO_2_@PDMS after calcination at 400 °C, and A-SiO_2_/N-TiO_2_@PDMS after three cycles of UV irradiation and calcination. Figure 10b–f show the narrow-scan spectra of O 1s in the above-mentioned spectra. Comparing the peak of C 1s, the intensity of C 1s peak was the highest in the A-SiO_2_/N-TiO_2_@PDMS spectrum, which was caused by the large amount of C bond in PDMS. After UV irradiation, the C 1s peak did not change significantly, but it decreased dramatically after calcination and three cycles of UV irradiation and calcination, indicating that C-H or C-Si bonds were fractured during the process. The change in the C 1s peak was consistent with the result of the FT-IR. Comparing the Ti 2p peaks, their intensity increased slightly after UV irradiation, which might be due to the generation of new Ti-containing bonds after UV irradiation. Moreover, it further increased in the spectra of A-SiO_2_/N-TiO_2_@PDMS after calcination and A-SiO_2_/N-TiO_2_@PDMS after three cycles of UV irradiation and calcination. This illustrates that new Ti-containing bonds might be generated after calcination. Meanwhile, more N-TiO_2_ was exposed, and the intensity of the Ti 2p peak was further enhanced due to the thermal decomposition of some C bonds. By comparing the peaks of O 1s, it was noted that the intensity of peaks did not significantly change before or after UV irradiation. However, the intensity of the O 1s peak notably increased in the spectra of A-SiO_2_/N-TiO_2_@PDMS after calcination and three cycles of UV irradiation and calcination. The reason for this phenomenon is similar to that of the strengthening of the Ti 2p peak mentioned above, i.e., the generation of new O-containing bonds and the thermal decomposition of some C-containing bonds might result in the exposure of more N-TiO_2_ and A-SiO_2_ on the surface.

Figure 10b shows that, in the spectrum of A-SiO_2_/N-TiO_2_, O 1s could be divided into two parts, which were caused by Ti-O-Ti and Si-O-Si. Furthermore, the peak at 529.9 eV was much higher than that of 532.4 eV. This is because A-SiO_2_ was covered by N-TiO_2_. Figure 10c shows the spectrum of A-SiO_2_/N-TiO_2_@PDMS. The Si-O-Si peak was much higher than that of Ti-O-Ti, which is because a considerable amount of Si-O-Si existed in the PDMS. Figure 10d shows the O 1s peak of A-SiO_2_/N-TiO_2_@PDMS after UV irradiation. The intensity of the O 1s peak at around 530 eV increased after UV irradiation and this peak could be divided into two peaks at 530.1 eV and 530.5 eV, which were attributed to Ti-O-Ti and Ti-O-Si, respectively. It was demonstrated that the Ti-O-Si bond was formed by the reaction of N-TiO_2_ and PDMS under UV irradiation [20]. The spectrum of A-SiO_2_/N-TiO_2_@PDMS after 400 °C calcination is shown in Figure 10e. The intensity of the O 1s peak (binding energy 532.4 eV) corresponding to the Si-O-Si bond was lower than that before calcination. It was attributed to the thermal decomposition of PDMS and the hydrolysis of PDMS to form silanol groups. The intensity of the O 1s peak corresponding to Ti-O-Ti and Ti-O-Si bonds was higher than that before calcination. This meant that PDMS could react with N-TiO_2_ on the surface of the coating to form Ti-O-Si bonds during the calcination process. Additionally, more N-TiO_2_ was exposed on the surface because of the thermal decomposition of some PDMS. As a result, the intensity of the Ti-O-Ti bond also significantly increased. In Figure 10f, the intensity of the O 1s peak corresponding to the Si-O-Si bond further decreased in the spectrum of A-SiO_2_/N-TiO_2_@PDMS after three cycles of UV irradiation and calcination. Moreover, the intensity of the O 1s peak corresponding to Ti-O-Ti and Ti-O-Si bonds, especially the intensity of the O 1s peak corresponding to Ti-O-Ti, further increased. This is because the thermal decomposition of PDMS tended to be complete, and the hydrophobic groups were exhausted. More N-TiO_2_ was exposed on the surface, which further strengthened the Ti-O-Ti bond.

### 3.6. Mechanism

**Figure 11:**
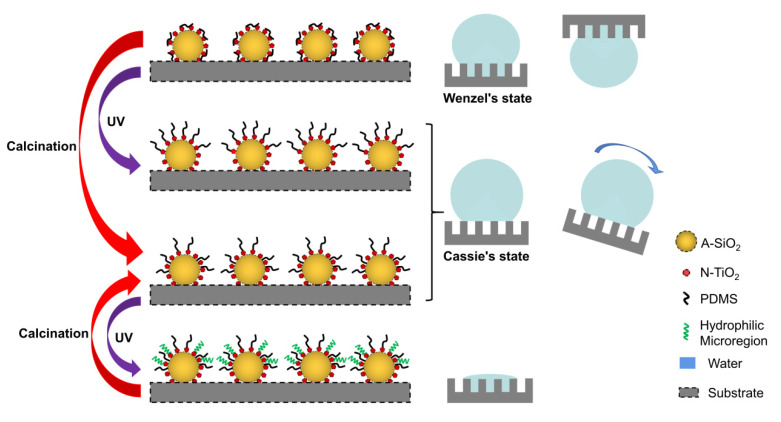
Wetting state and surface microstructure of the A-SiO_2_/N-TiO_2_@PDMS coating under different conditions.

In this study, the mechanism of the self-cleaning property of an as-prepared coating was analyzed according to the experimental phenomena, test results, and existing research results. Figure 11 shows the microstructure, wetting state, and reaction mechanism of the coating after UV irradiation and calcination at 400 °C.

Firstly, the mechanism of the superhydrophobicity and high adhesion of the coating is as follows: The superhydrophobicity of the coating was caused by the hierarchical structure of A-SiO_2_/N-TiO_2_ and the low surface energy of PDMS [24,25]. The adhesion of its surface was due to the fact that A-SiO_2_/N-TiO_2_ might not be covered by PDMS completely, and a small part of -OH was exposed on the surfaces. The hydrophilicity of -OH resulted in high adhesion to water droplets [26]. At this time, the surface of the coating was in Wenzel’s state.

Secondly, the mechanism of the increase in superhydrophobicity and decrease in adhesion of the coating after UV irradiation is as follows: N-TiO_2_ produced electron–hole pairs under UV irradiation. Then, the electron–hole pairs may have excited the generation of hydroxyl groups and water molecules. The activated molecules partially cleaved siloxane bonds of PDMS. These segmented siloxane-based chains formed a covalent bond with N-TiO_2_ via a Ti-O-Si bond, resulting in the occupation of hydroxyl groups and the existence of more hydrophobic groups on the coating surface [23]. At this time, the surface of the coating was in Cassie’s state. The reaction mechanism is illustrated in Figure S2.

Thirdly, the mechanism of the increase in superhydrophobicity and decrease in adhesion of the coating after calcination at 400 °C is discussed below. At this time, the surface of the coating was still in Cassie’s state. The mechanism is analyzed as follows: water molecules on the surface reacted with PDMS, and PDMS hydrolyzed to form silanol groups which condensed with the hydroxyl groups of A-SiO_2_ and N-TiO_2_, thus forming Si-O-Si and Si-O-Ti bonds [27]. This caused PDMS to be grafted onto the A-SiO_2_ and N-TiO_2_ surfaces. The mechanism is shown in Figure S3. A part of the decomposed PDMS formed Si-O-Si bonds with SiO_2_, so the Ti hydroxyl groups on the coating surface were not completely occupied. This led to the exposure of some N-TiO_2_ on the surface. This is the reason why the calcined A-SiO_2_/N-TiO_2_@PDMS coating surface lost its superhydrophobicity after UV irradiation.

Fourth is the mechanism of the reversible wettability of the coating after UV irradiation and calcination. As mentioned above, PDMS could form covalent bonds with A-SiO_2_ and N-TiO_2_ via Si-O-Si and Si-O-Ti bonds under calcination at 400 °C. However, some Ti hydroxyls might not have reacted with PDMS, and they were exposed on the surface. Electron–hole pairs were generated on the surface of N-TiO_2_ under UV irradiation. The electron reacted with Ti^4+^, and the hole reacted with the surface bridging oxygen, forming Ti^3+^ and oxygen vacancy, respectively. At this time, water in the air was absorbed by the oxygen vacancies and became chemisorbed water. The chemisorbed water further adsorbed moisture in the air to form hydrophilic micro-regions around Ti^3+^ defects. The water on the surface was absorbed by the hydrophilic micro-regions, so the surface of the coating was hydrophilic [28]. After calcination at 400 °C for 2 h, the wettability of the as-prepared coating changed from hydrophilicity to superhydrophilicity. This is because the chemisorbed hydroxyl was replaced by oxygen in the air after calcination, and the hydrophilic micro-regions disappeared. The wettability of the as-prepared coating could not change from hydrophilicity to superhydrophilicity after three cycles of UV irradiation and calcination, because PDMS was exhausted during the process of repeated UV irradiation and calcination, and the hydrophobic groups almost completely disappeared. Eventually, the A-SiO_2_/N-TiO_2_@PDMS coating showed a hydrophilic state.

## 4. Conclusions

In conclusion, cheap and nontoxic A-SiO_2_ composites were selected as carriers, and the A-SiO_2_/N-TiO_2_ composites were prepared by the hydrophobic aggregation method. Then, the A-SiO_2_/N-TiO_2_ composites were modified with PDMS. Finally, the intelligent superhydrophobic A-SiO_2_/N-TiO_2_@PDMS coating was formed by the spraying method. The WCA of the coating was further enhanced, and its CAH was decreased after UV irradiation and calcination at 400 °C. The A-SiO_2_/N-TiO_2_@PDMS was suitable for constructing self-cleaning coatings on the surfaces of different types of substrates, such as glass, wood, foam, concrete, and brick. Moreover, the coating maintained self-cleaning properties after being placed outdoors for 3 months, showing a good adaptability to the outdoor environment. Therefore, the A-SiO_2_/N-TiO_2_@PDMS could be used to construct a self-cleaning coating with superhydrophobicity, low adhesion to water droplets, and photocatalytic activity under ultraviolet or sunlight irradiation. The coating not only had the excellent self-cleaning properties but also the advantages of cheap and readily available raw materials, a simple preparation process, being pollution-free, and broad practical application prospects.

## Supplementary Materials

The following are available online at https://www.mdpi.com/article/10.3390/nano11061486/s1, Table S1: The wettability and adhesion force of the A-SiO2/N-TiO2@PDMS coating to water droplets before and after UV/sun irradiation and calcination, Table S2: CAs of the surfaces of a glass slide and the A-SiO2/N-TiO2@PDMS coating after UV irradiation to different droplets, Figure S1: TG curves of A-SiO2/TiO2 and A-SiO2/TiO2@PDMS, Figure S2: Reaction mechanism of PDMS and N-TiO2 under UV irradiation, Figure S3: Reaction mechanism of PDMS with N-TiO2 and A-SiO2 under calcination at 400 °C.
