# Supplementary material for: Tunable Adhesive Self-Cleaning Coating with Superhydrophobicity and Photocatalytic Activity
Source: Nanomaterials (Basel). 2021 Jun 3;11(6):1486. doi: 10.3390/nano11061486 (PMC8229519; doi:10.3390/nano11061486)
Supplement: Supplementary file 1 [file nanomaterials-11-01486-s001.zip › nanomaterials-1210526-supplementary.pdf]

nanomaterials

MDPI

Supplementary material

Tunable Adhesive Self-Cleaning Coating with Superhydrophobicity and Photocatalytic Activity

Xuan Wang, Hao Ding *, Sijia Sun, Han Zhang, Run Zhou, Yangzi Li, Jie Wang and Weihua Ao *

Beijing Key Laboratory of Materials Utilization of Nonmetallic Minerals and Solid Wastes, National Laboratory of Mineral Materials, School of Materials Science and Technology, China University of Geosciences, Xueyuan Road, Haidian District, Beijing 100083, China; wangxuan0505@163.com (X.W.); sunsijia@cugb.edu.cn (S.S.); zhanghan0050@163.com (H.Z.); 3003180004@cugb.edu.cn (R.Z.); liyangzi@cugb.edu.cn (Y.L.); 3003170004@cugb.edu.cn (J.W.)

* Correspondence: dinghao113@126.com (H.D.); awh0223@cugb.edu.cn (W.A.)

Experimental details

Measurement of WCAs and CAHs

The wettability of surfaces was characterized by WCAs. The adhesion of surfaces to water droplets was characterized by CAH. WCAs and CAHs were determined by a contact angle meter (JC2000D, Shanghai Zhongchen Digital Technic Apparatus Co., Ltd., China), and the volume of droplets was 8 μL. Moreover, WCAs and CAHs were determined by measuring each sample at five diverse positions.

Measurement of the surface adhesive force to water droplets

The surface adhesive force to water droplets was measured by a highly sensitive dynamic contact angle detector (DCAD, Dataphysics, Germany). A 8 μL water droplet was suspended with a metal ring first, and the coating was placed on the balance table. The water droplet was moved downward at a constant speed of 0.01 mms^{-1} until the water droplet came into contact with the surface of the coating. Then, the water droplet was moved upward, and the force increased gradually until it reached its maximum. Each sample was measured five times, and an average value of adhesion was given.

UV treatment of the A-SiO_2/N-TiO_2@PDMS coating

A 500 W high-pressure mercury lamp (model: XE-JY500, wavelength: 365 nm, irradiation intensity: 81 mW/cm^2) from Beijing Newbit Technology Co., Ltd, Beijing, China was used to irradiate the A-SiO_2/N-TiO_2@PDMS coating for ultraviolet light treatment, and the distance between the light source and the as-prepared coating was about 20 cm.

Evaluation of photocatalytic activities

The photocatalytic activities of A-SiO_2/N-TiO_2@PDMS and other samples were evaluated by the degradation of methyl orange dilution under UV light. The degradation experiments were performed in a cylindrical quartz tube under magnetic stirring at 25°C. The 50 mg samples were added to 50 mL of prepared methyl orange dilution with a concentration of 10 mg/L (C_0). The above-mentioned suspensions were stirred for 1 h in the dark to reduce the measurement error caused by sample adsorption. After turning on the 500 W high-pressure mercury lamp, the photoreaction started. The suspensions were taken and centrifuged at a given time, and then the absorbance to 464 nm light of the supernatant was measured with a Cary 5000 UV-VIS spectrophotometer (USA Varian,

USA). The absorbance was converted into the concentration of methyl orange at irradiation time t (C). The photocatalytic degradation performance of samples was characterized by C/C_0.

Characterizations

The morphologies of A-SiO_2, N-TiO_2, and A-SiO_2/N-TiO_2 composites were observed by scanning electron microscope (SEM) (S-3500N, HITACHI, Japan). The functional groups on the surfaces of the samples were detected by Fourier transform infrared (FT-IR) (Spectrum 100, PerkinElmer Instruments (Shanghai) Co., Ltd., Shanghai, China), using KBr as the medium. The weights of each sample and KBr were 3 and 130 mg, and the scanning range was 4000-400 cm^{-1}; The crystal structures of the samples were characterized by X-ray diffractometer (XRD) (D8 ADVANCE, BrukerAXS GmbH, Karlsruhe, Germany) with Cu Kα radiation (λ=1.5406 Å) generated at 40 kV and 40 mA. The type of elements, relative content, and energy level structure between samples were measured by X-ray photoelectron spectroscopy (XPS) (Thermo Fisher Scientific USA) with a monochromatized Al Kα line source (200 W). The thermal behaviours were evaluated using a TG thermal analyser (STA499, NETZSCH, Germany).

Table S1. The wettability and adhesion force of the A-SiO_2/N-TiO_2@PDMS coating to water droplets before and after UV/sun irradiation and calcination.

Treatment of A-SiO_2/N-TiO_2@PDMS coating	Un-treated	UV irradiation for 1 min	UV irradiation for 8 h	Sun irradiation for 1 min	Calcination at 400°C for 30 min	Calcination at 400°C for 30 min and UV irradiation for 2 h	Calcination at 400°C for 30 min and sun irradiation for 2 h
Water contact angle/°	151.2	158.0	158.1	158.3	159.7	19.1	21.2
Water sliding angle/°	---	3.4	3.2	3.1	2.3	---	---
Adhesion force/μN	109.9	10.4	---	---	---	---	---

Table S2. CAs of the surfaces of a glass slide and the A-SiO_2/N-TiO_2@PDMS coating after UV irradiation to different droplets.

Different droplets	Water	HCl droplet	NaOH droplet	Methyl orange droplet
Glass slide	45.6°	41.2°	23.5°	43.1°
A-SiO_2/N-TiO_2@PDMS after UV irradiation for 1 min	158.0°	156.4°	153.8°	155.8°

Thermal stability of the A-SiO_2/N-TiO_2@PDMS coating

To understand the thermal stability of the as-prepared coating, TG analysis of A-SiO_2/N-TiO_2 and A-SiO_2/N-TiO_2@PDMS was carried out, and the results are shown in Figure S1. In the TG curve of A-SiO_2/N-TiO_2, the weight loss of 3.0% in the temperature range of 25-800°C was ascribed to the evaporation of adsorbed water and the decomposition of residual organic matter and other impurities. The TG curve of A-SiO_2/N-TiO_2@PDMS showed a weight loss of 18.4% in the temperature range of 25-800°C, which was mainly caused by the thermal decomposition of PDMS. The TG curves of A-SiO_2/N-TiO_2 and A-SiO_2/N-TiO_2@PDMS changed slightly in the temperature range of 25-250°C. During this period, a small weight loss occurred, which was mainly attributed to the evaporation of adsorbed water. When the temperature was higher than 250°C, the weight loss of A-SiO_2/N-TiO_2@PDMS was more obvious. The weight loss in the range of 250-500°C was mainly caused by the fracture and oxidative decomposition of partially branched chains.

Then, PDMS completely decomposed in the range of 500-670°C. In the temperature range of 670-800°C, the remaining A-SiO_2/N-TiO_2 tended to be stable. The results showed that when the temperature was lower than 500°C, PDMS was almost stable. Therefore, the A-SiO_2/N-TiO_2@PDMS coating has good thermal stability in the temperature range of 25-500°C.

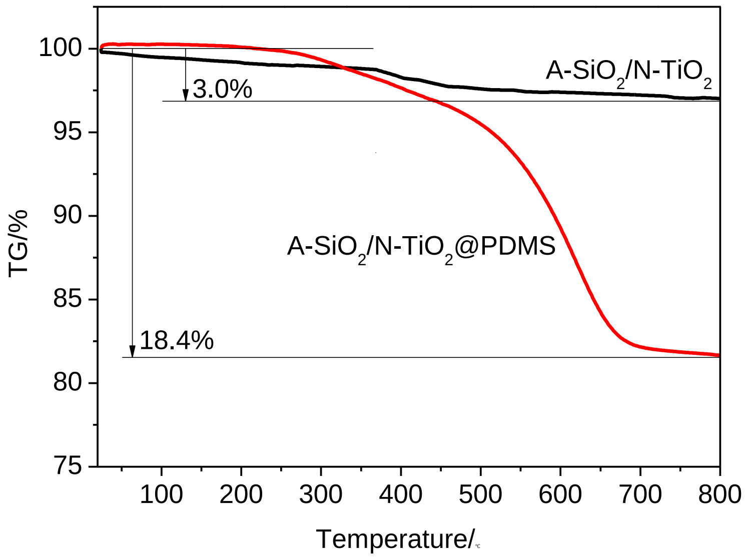

Figure S1. TG curves of A-SiO_2/TiO_2 and A-SiO_2/TiO_2@PDMS.

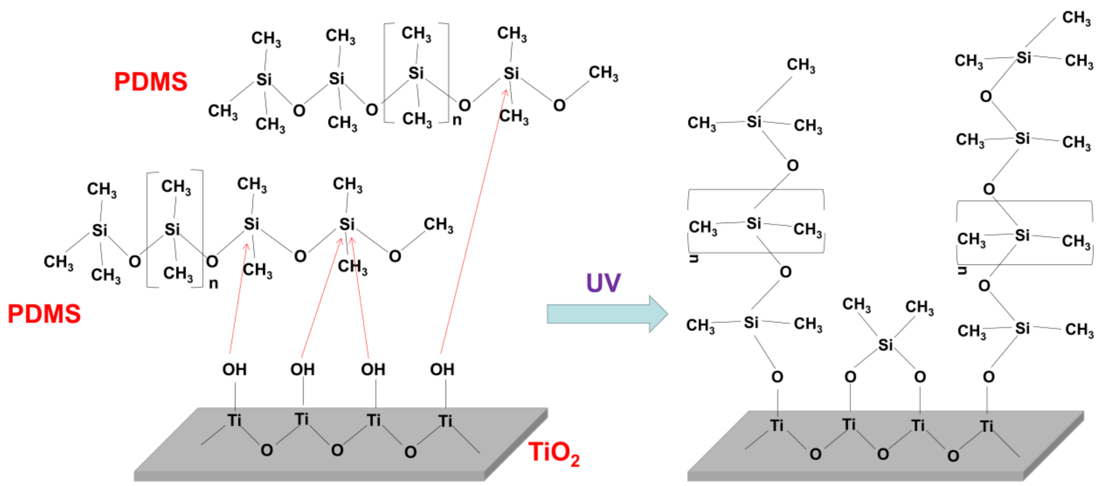

Figure S2. Reaction mechanism of PDMS and N-TiO_2 under UV irradiation.

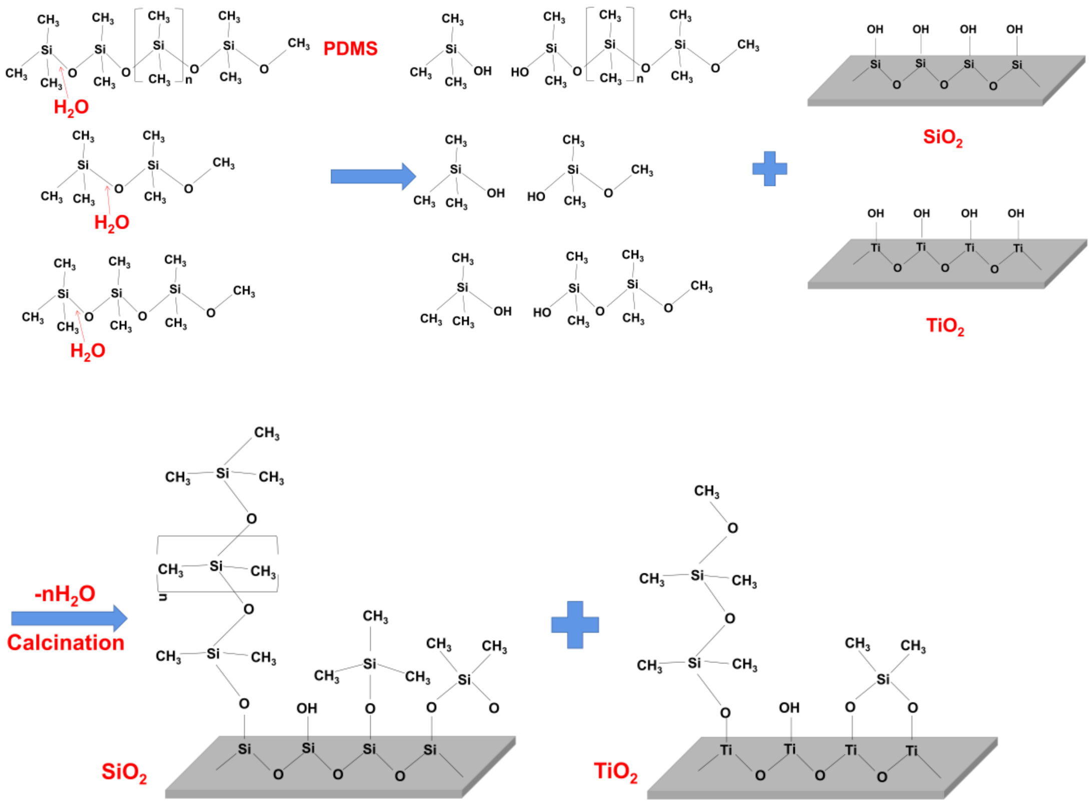

Figure S3. Reaction mechanism of PDMS with N-TiO_2 and A-SiO_2 under calcination at 400°C.
